# Supplementary fixation in multifragmentary midshaft clavicle fractures: do interfragmentary screws or suture cerclage improve outcomes compared with plate-only fixation?

**DOI:** 10.1007/s00068-026-03248-8

**Published:** 2026-06-16

**Authors:** Christiane Barthel, Michelle Antonia Hinkelmann, Desiree Schibler, Flurina Frei, Hans-Christoph Pape, Florin Allemann

**Affiliations:** 1 Department of Shoulder and Elbow Surgery, Schulthess Klinik, Lengghalde 2, Zurich, CH-8008 Switzerland; 2 Department of Traumatology, University Hospital, Zurich, Switzerland; 3 University of Zurich, Zurich, Switzerland

**Keywords:** Multifragmentary clavicle fracture, Open reduction and internal fixation, Plate-independent screws, Suture cerclage, Interfragmentary screw, Callus formation

## Abstract

**Background:**

Clavicle fractures are common injuries. As displaced or multifragmentary midshaft patterns carry a substantial risk of malunion and nonunion with nonoperative treatment, they have contributed to the increasing use of surgical fixation. Modern operative techniques include plate fixation, intramedullary devices, and hybrid strategies to address comminution and improve fragment stability. Supplementary interfragmentary screws and cerclage constructs have been proposed to enhance fragment control and optimize load transfer in multifragmentary fracture patterns. This study aimed to compare three fixation strategies for operatively treated midshaft clavicle fractures managed with plate osteosynthesis: Group IS (interfragmentary screw), Group CA (cerclage augmentation), and Group POF (plate-only fixation), with respect to operative time, complication rates, and potential biological and mechanical differences.

**Methods:**

This retrospective study included patients aged ≥ 16 years who underwent plate fixation for multifragmentary clavicle fractures between 2016 and 2024. Patients were categorized into three groups: Group IS (supplementary interfragmentary screw), Group CA (suture cerclage augmentation), and Group POF (plate-only fixation). Demographic, surgical, and radiographic data were collected. Callus formation was assessed by three independent observers. Statistical analyses were performed with a significance level of *p* < 0.05.

**Results:**

A total of 246 patients (mean age 44.5 ± 15.7 years; 89% male) were included. The overall complication rate was 6.9%. Fracture complexity differed significantly between groups (*p* = 0.0083), with Group IS and Group CA including more complex fracture patterns. Operative time was longest in Group CA (101.1 min), followed by Group IS (90.1 min) and Group POF (82.4 min; p < 0.001). Group IS and Group CA demonstrated lower complication rates (6.0% and 5.5%, respectively) compared with Group POF (16.3%, p = 0.0444). Mechanical complications were absent in Group CA (p = 0.035). Differences in callus formation were observed between groups (p = 0.0059). Refracture rates after implant removal were highest in Group CA (16.0%), with no refractures observed in Group POF (p = 0.064).

**Conclusion:**

While the choice between interfragmentary screws and cerclage is primarily dictated by fracture morphology, our findings suggest that supplementary fixation may be beneficial in multifragmentary fractures. Both strategies were associated with lower complication rates compared with plate-only fixation; however, refracture rates after implant removal may be higher and require cautious interpretation. Further studies are needed to clarify the long-term clinical impact of these strategies.

**Level of Evidence:**

Level III, Retrospective cohort study.

## Introduction

Clavicle fractures represent a substantial proportion of skeletal injuries and may lead to persistent functional limitations when displaced or comminuted. They account for approximately 5–10% of all fractures and 35–45% of shoulder-girdle injuries, with the midshaft region being the most commonly affected [1]. Displaced or multifragmentary midshaft fractures carry a substantial risk of symptomatic malunion and nonunion after nonoperative treatment, with reported nonunion rates of up to 15–20% [2, 3], prompting increased consideration of surgical fixation in selected patients [1, 4]. Contemporary trends reflect a growing use of operative management to mitigate loss of alignment, symptomatic shortening, and delayed return to function associated with nonoperative care and to enable earlier rehabilitation in some populations [5].

Operative strategies have evolved considerably. Whereas most midshaft fractures were historically treated nonoperatively, the recognition of poorer outcomes in selected displaced patterns has led to broader adoption of surgical fixation [5]. Precontoured superior or anterior 3.5-mm plates and locking compression plates remain widely used to restore length, alignment, and rotational stability [6, 7].

Intramedullary devices—including elastic nails and cannulated screws—have been introduced as lower-profile options with reduced soft-tissue disruption, and newer implants have undergone focused biomechanical evaluation [8–10].

Hybrid strategies have also gained attention, with combinations such as plate fixation plus interfragmentary screws or dual-plate constructs used to address comminution, improve fragment control, or reduce implant prominence and reoperation rates [5, 6, 11]. Cerclage techniques are particularly useful for stabilizing multiple small fragments when screw purchase is limited, offering improved fragment capture, potentially shorter operative times and reduced fluoroscopy [12, 13]. Supplementary interfragmentary or cortical screws aim to improve fragment apposition and modify load transfer to make plate constructs more predictable under physiological loading.

Finite-element models demonstrate that lag-screw compression reduces motion at the fracture site, lowers shear forces, and decreases stresses on the plate [14].

Cadaveric and sawbone experiments demonstrate that adding an anterior mini‑plate or using dual‑plate/orthogonal constructs increases torsional and bending stiffness compared with single superior plating, which implies that buttressing fragments in multiple planes resists gap motion [6, 15, 16]. However, while biomechanical studies have provided insight into these techniques, there remains a gap between research findings and clinical outcomes. Further investigation is necessary to determine functional outcomes and complication rates associated with each fixation method.

This study aims to address this gap by evaluating the clinical and radiographic outcomes of three distinct fixation strategies: plate-independent screw fixation, suture cerclage augmentation, and plate-only constructs, with the goal of identifying any biological or mechanical advantages associated with each approach.

## Materials and methods

This retrospective single-center study included all patients surgically treated for clavicle fractures at the University Hospital Zurich between 2016 and 2024. Inclusion criteria were age ≥ 16 years, written informed consent, a multifragmentary fracture pattern with ≥ 3 fragments, and operative treatment with plate fixation. Exclusion criteria were lack of consent, nonoperative management, absence of postoperative follow-up, or simple two-part fractures.

**Fig. 1:**
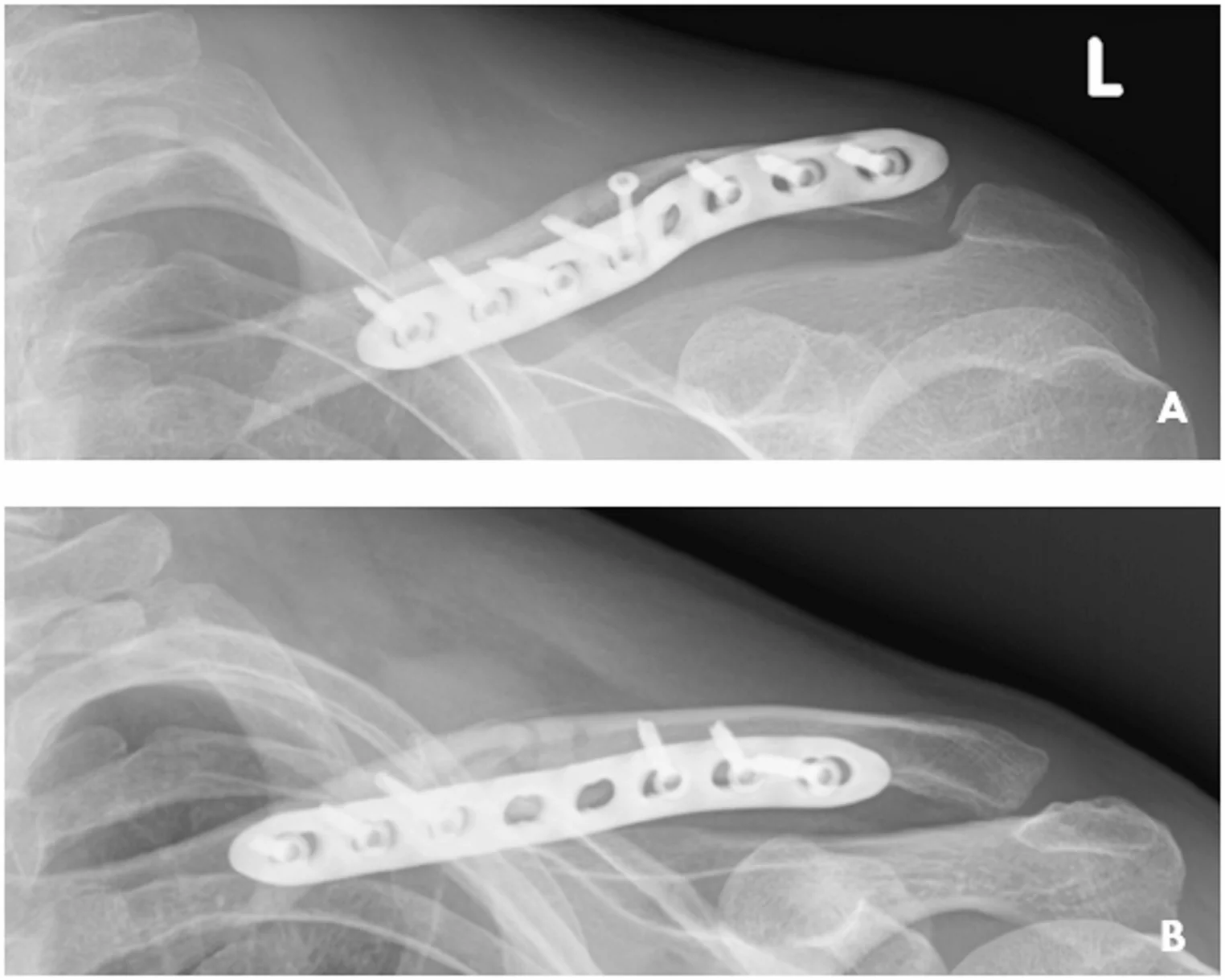
Representative radiographs of multifragmentary midshaft clavicle fractures treated with different fixation strategies. **(A)** Plate fixation with supplementary interfragmentary screw. **(B)** Plate-only fixation

Patients were allocated to three groups based on their fixation strategy: Group IS (plate fixation with supplementary plate-independent interfragmentary screws, applied either as classical lag screws according to AO principles or as cortical augmentation screws to maintain fragment apposition without generating compression), Group CA (plate fixation with supplementary suture cerclage), and Group POF (plate fixation without additional fixation) (Fig. 1).

Surgical details, implant configurations, and clinical follow-up data were obtained from our clinical information system. Extracted variables included patient demographics, fracture morphology, surgical technique, implant characteristics (plate type, screw distribution, suture cerclage use), and implant removal. Fractures were classified using a pre-2018 AO system and grouped for analysis to describe relative morphology and complexity. Multifragmentation was defined independently as ≥ 3 fragments on preoperative imaging. Complications were defined as events requiring additional treatment. Persistent pain was defined as pain interfering with daily activities requiring treatment, and impaired wound healing as delayed wound healing requiring additional management in the absence of infection. Radiographic follow-up adhered to institutional standards, and fracture healing was assessed by evaluating callus formation at predefined intervals. Inter- and intra-observer reliability for radiographic callus assessment was evaluated by three independent observers, with one observer repeating assessments after three months. Fracture healing was further evaluated through biomechanical analysis by recording refracture rates following implant removal.

Statistical analyses were performed using Python™ version 3.10. Inter- and intra-observer agreement was assessed using Cohen’s kappa coefficient with 95% confidence intervals. Descriptive statistics (means and standard deviations) summarized demographic and clinical variables. Categorical variables were compared using Chi-square or Fisher’s exact tests, while continuous variables were analyzed using T-tests or Mann–Whitney U tests, depending on distribution. For comparisons involving more than two groups, the Kruskal–Wallis test was applied, followed by Bonferroni-adjusted post-hoc pairwise comparisons where appropriate. Statistical significance was set at *p* < 0.05.

## Results

A total of 246 patients were included (mean age 44.5 ± 15.7 years; median 43; range 17–90), 89.0% of whom were male. Laterality was balanced (49.6% left, 50.4% right). Fracture morphology was described using grouped categories derived from a detailed pre-2018 AO classification, with 162 patients (65.9%) assigned to B2, 72 (29.3%) to B3, and 12 (4.9%) to B1 (Table 1).

**Table 1:** Fracture morphology categories (Grouped from a Pre-2018 AO Classification)

Fracture morphology	Group IS (*n* = 107)	Group CA (*n* = 74)	Group POF (*n* = 65)
B1	4 (3.7%)	0 (0%)	8 (12.3%)
B2	76 (71.0%)	48 (64.9%)	38 (58.5%)
B3	27 (25.2%)	26 (35.1%)	19 (29.2%)

Patients were categorized into three cohorts: Group IS (interfragmentary screw) (*n* = 107), Group CA (cerclage augmentation) (*n* = 74), and Group POF (plate-only fixation) (*n* = 65). Baseline demographics were comparable across groups (age *p* = 0.303; sex *p* = 0.657). The Chi-square test revealed a significant difference in the distribution of fracture morphology categories between the groups (Chi-square = 13.70, *p* = 0.0083). Group IS and CA had a higher proportion of morphologically more complex fracture patterns (grouped B2/B3 categories) compared with Group POF. A manually contoured 3.5-mm LCP was used in nearly all procedures, except for one Group POF, two Group IS, and three Group CA cases treated with a 2.7/3.5-mm anterior–superior LCP.

Operative duration differed significantly (H = 17.15; *p* < 0.001), with the longest procedures in Group CA (101.1 min), followed by Group IS (90.1 min) and the shortest procedures in Group POF (82.4 min) (Table 2). Post-hoc testing confirmed that cerclage fixation required significantly longer operative times than both other groups (adjusted *p* = 0.009 and < 0.001), while the latter two did not differ (*p* = 0.538).

**Table 2:** Operative time by fixation strategy

Group	*n*	Mean OP time (min)	Median (min)	SD (min)	Min	Max
CA	74	101.1	101.5	26.5	44	170
POF	65	82.4	80	20.1	30	129
IS	107	90.1	87	26.5	38	178

The overall complication rate was 8.1% (20/246). Group-specific rates were 6.0% in Group IS, 5.5% in Group CA, and 16.3% in Group POF, with a significant difference between the groups (Chi-square = 6.23, *p* = 0.0444) (Table 3). Mechanical complications (implant failure, peri-implant fracture or nonunion) occurred in 0% of Group CA cases compared with 5.8% in all other patients combined, representing a significant risk reduction (*p* = 0.035; ARD − 5.8%, 95% CI − 9.3% to − 2.3%) (Table 4).

**Table 3:** Overall complication rates by fixation strategy

Group	Patients (*n*)	Complications (*n*)	Complication rate (%)	*p*-value
IS	107	6	6	
CA	74	4	5.5	
POF	65	10	16.3	0.044

**Table 4:** Complication profile by type and fixation strategy

Group	implant mal- position	surgical site infection	implant failure	peri-implant fracture	Non-union	persistent pain	impaired wound healing	Total (%)
CA	0	1.4	0	0	0	2.7	0	5.5
POF	0	1.5	4.6	1.5	1.5	0	1.5	16.3
IS	0.9	0	2.8	0.9	0.9	0.9	0	6

Callus formation differed significantly among the three groups (Chi² = 10.26; *p* = 0.0059) (Table 5). Group CA demonstrated the highest callus rate (93.2%), followed by Group POF (86.2%), while Group IS showed the lowest (75.7%). Post-hoc analysis showed differences between groups, with lower callus formation in Group IS compared with Group CA (adjusted *p* = 0.0068), as expected based on the underlying fixation principles. Intraobserver agreement was moderate (unweighted Cohen’s κ = 0.45; weighted κ = 0.33). Interobserver reliability across the three raters was also moderate (Fleiss’ κ = 0.48), with full agreement achieved in 46.4% of cases.

**Table 5:** Radiographic callus formation by fixation strategy

Group	Callus *n* (%)	95%-CI (%)	No Callus *n* (%)	Total
CA	69 (93.2)	85.1–97.1	5	74
POF	56 (86.2)	75.7–92.5	9	65
IS	81 (75.7)	66.8–82.8	26	107

Refracture rates after implant removal were highest in Group CA (16.0%), followed by Group IS (5.1%), and 0% in Group POF, though the overall difference did not reach significance (*p* = 0.10). When comparing Group CA with all other strategies combined, the difference approached significance (*p* = 0.064), corresponding to an odds ratio of 5.33 (95% CI ≈ 0.91–31.31) and an absolute risk increase of 12.6% (95% CI − 2.6% to + 27.7%). Time to implant removal was comparable between groups (IS: 21.1 ± 5.5 months, CA: 22.9 ± 8.2 months, POF: 21.7 ± 9.2 months) (Table 6).

**Table 6:** Implant removal outcomes

Group	*n*	Refracture *n* (%)	Time to removal (months)
IS	39	2 (5.1%)	21.1 ± 5.5
CA	25	4 (16.0%)	22.9 ± 8.2
POF	19	0 (0%)	21.7 ± 9.2
p-value (overall)	—	0.1	0.74

## Discussion

This retrospective study compared supplementary fixation strategies— Group IS (plate-independent interfragmentary screws), Group CA (suture cerclage), and Group POF (plate-only constructs)—in the treatment of multifragmentary midshaft clavicle fractures. Differences were observed in operative efficiency, overall complication rates and healing patterns, providing practical guidance for fixation strategy selection.

### Operative time

In the three-way comparison, Group CA was associated with the longest operative times. This may reflect the technical demands of cerclage placement, particularly around comminuted fragments [12, 13], as well as greater fracture complexity and the associated need for more demanding reduction in cases where cerclage is selected. As described for the Nice knot technique, operative time may shorten with experience due to ease of fragment capture and reduced need for fluoroscopy [12].

### Complications and construct behavior

Overall complication rates differed significantly between groups. The Group POF showed the highest complication rate, although individual major complication categories were infrequent, possibly reflecting selection bias or limited stability in fractures with unrecognised instability. In contrast, no mechanical complications occurred in Group CA, compared with a 5.8% rate in Groups IS and POF combined. This supports prior reports of high mechanical reliability in cerclage-augmented constructs: Wu et al. found 100% union with no implant failures in 56 patients treated with double-suture Nice knots [13], and Lee et al. reported universal union in 116 patients treated with cerclage wire augmentation [17]. These results suggest that cerclage may distribute loads more favorably than rigid screw constructs in highly comminuted fracture patterns.

### Callus formation and healing patterns

Differences in callus formation were observed between fixation strategies, with lower callus formation in Group IS, consistent with the underlying biomechanical environment (Group CA > Group POF > Group IS). This aligns with biomechanical data showing that lag screw compression reduces interfragmentary motion and promotes more primary bone healing with limited periosteal callus [14, 18]. Importantly, despite differing radiographic healing patterns, no statistically significant differences in refracture rates after implant removal were observed; however, the numerically higher rate in the cerclage group warrants cautious interpretation given the limited number of events. Both primary and secondary bone healing can result in successful union [5, 19].

Cerclage fixation, which may allow controlled micromotion and preservation of fragment continuity, was associated with robust callus formation without delayed union [5, 17]. This supports previous findings showing unchanged union times regardless of cerclage use and contradicts concerns about impaired periosteal perfusion [17].

### Refracture risk after implant removal

Refracture rates after implant removal were higher in Group CA compared with Group IS and Group POF, representing a clinically relevant finding, although the difference did not reach statistical significance. A post-hoc power analysis (Group CA vs. all other strategies combined) yielded a power of 46.8%, indicating that the analysis was underpowered to detect clinically relevant differences. Given the limited number of events and wide confidence intervals, these findings should be interpreted with caution, and the absence of statistical significance should not be considered evidence of equivalence. The observed pattern may suggest that cerclage constructs do not always achieve complete cortical remodeling before hardware removal. Circumferential compression and residual stress risers after cerclage removal, as well as abundant callus reflecting persistent micromotion, may contribute [13, 20, 21]. Clinically, these findings support cautious timing of implant removal after cerclage fixation, ideally after radiographic confirmation of complete cortical bridging, and appropriate patient counseling.

Interpretation and Future Directions

The observed differences in operative duration, biological healing patterns, and refracture tendencies highlight that supplementary fixation modifies both the mechanical environment and the mode of fracture healing. Screw augmentation increases construct rigidity and reduces interfragmentary motion [14, 18], which may explain the lower callus volumes observed in this group. Cerclage fixation, by contrast, appears to permit controlled micromotion that stimulates abundant periosteal callus formation [17] and provides reliable fragment control, despite prolonged operative time. Given that fracture morphology often dictates the choice between cerclage and interfragmentary screws, the most relevant finding of this study is that additional fixation, rather than bridging alone, is associated with lower complication rates.

Future prospective studies incorporating direct biomechanical testing, advanced imaging to evaluate cortical consolidation, and validated patient-reported outcome measures are needed to clarify how these differing stability profiles and healing patterns translate into functional recovery and long-term structural integrity.

## Limitations

Several limitations should be considered when interpreting the findings of this study. First, the retrospective design introduces potential biases, particularly in patient selection and surgeon decision-making regarding fixation strategies. As treatment allocation was not randomized and fracture complexity differed significantly between groups, the observed differences in complication rates may be influenced by confounding and surgeon selection bias. Supplementary fixation may have been preferentially applied in more complex fractures where improved biomechanical stability was anticipated. Therefore, the observed differences cannot be causally attributed to the fixation strategy alone.

In addition, the refracture analysis was underpowered, as reflected by a post-hoc power of 46.8%, limiting the ability to detect clinically relevant differences. Detailed information regarding the exact trauma mechanism leading to refracture after implant removal was not consistently available due to the retrospective design. It therefore cannot be determined with certainty whether these events represent true post-removal refractures or incomplete biological healing. Cases occurring after minor or no trauma may instead reflect unrecognised nonunion and would ideally be classified as complications. Future prospective studies should systematically document refracture mechanisms to allow more accurate categorisation and complication reporting.

Most cases in this cohort were treated using manually contoured straight 3.5-mm locking compression plates, reflecting institutional practice during the study period. Contemporary precontoured low-profile clavicle plates (e.g., 2.7–3.5 mm) may provide improved implant fit and reduced soft-tissue irritation, potentially lowering implant-related complications or implant removal rates [16, 22]. Therefore, the direct applicability of our findings to current implant systems may be limited.

Moreover, procedures were performed by surgeons with varying levels of experience, which may have introduced variability in surgical technique and outcomes. Intra- and inter-rater reliability for radiographic assessment of callus formation were moderate, indicating potential measurement variability that may have influenced the observed differences between groups. Finally, functional outcomes, such as range of motion and patient-reported outcomes, were not assessed, limiting conclusions regarding recovery and long-term clinical relevance.

## Conclusions

This study compared supplementary fixation strategies—plate-independent interfragmentary screws, suture cerclage, and plate-only constructs—in the treatment of multifragmentary midshaft clavicle fractures. Cerclage fixation was associated with longer operative times. Both supplementary fixation strategies were associated with lower complication rates compared with plate-only fixation, suggesting that additional fragment stabilization may be beneficial in multifragmentary fractures, although causality cannot be established due to potential confounding.

Screw augmentation was associated with reduced radiographic callus formation, consistent with its more rigid biomechanical behavior. Refracture rates after implant removal showed no statistically significant difference; however, these findings require cautious interpretation given the limited number of events, particularly in the cerclage group.

Taken together, these findings suggest that additional fixation may be associated with improved outcomes, with both techniques representing viable adjuncts to plate fixation depending on fracture morphology. Cerclage may be useful in highly comminuted fractures requiring fragment capture, whereas screw augmentation provides rigid interfragmentary stability without increasing operative morbidity. Further prospective studies incorporating biomechanical comparisons and patient-reported outcomes are warranted to better define the long-term clinical impact of these strategies.

## Data Availability

No datasets were generated or analysed during the current study.
